# Editorial: Plant Responses to Biotic and Abiotic Stresses: Lessons from Cell Signaling

**DOI:** 10.3389/fpls.2017.01772

**Published:** 2017-10-13

**Authors:** Sylvain Jeandroz, Olivier Lamotte

**Affiliations:** Agroécologie, AgroSup Dijon, Centre National de la Recherche Scientifique, Institut National de la Recherche Agronomique, Université Bourgogne Franche-Comté, Dijon, France

**Keywords:** abiotic stress, biotic stress, signaling, plants, adaptation, physiological

Facing stressful conditions imposed by their environment that could affect their growth and their development throughout their life cycle, plants must be able to perceive, to process, and to translate different stimuli into adaptive responses. Understanding the organism-coordinated responses involves fine description of the mechanisms occurring at the cellular and molecular level. These mechanisms involve numerous components that are organized into complex transduction pathways and networks, from signal perception to physiological responses. The major challenge of plant signaling is to understand how the large diversity of molecules identified as signals, sensors, or effectors could drive a cell to the appropriate plant response, to cope with various environmental challenges. The objective of this Research Topic is to give an overview of various signaling mechanisms or to present new molecular signals involved in stress response and to demonstrate how basic/fundamental research on cell signaling will help to understand stress responses at the whole plant level.

Under a changing climate, drought becomes one of the most critical abiotic stresses that severely reduces crop production. Molecular mechanisms involved in plant drought adaptation are addressed in several articles/contributions. Reversible protein phosphorylation, catalyzed by antagonistic activities of protein kinases and phosphatases, is a predominant molecular switch controlling the outcome of cell signaling after stress perception. Vilela et al. present an extensive review of the role of Casein Kinase 2 (CK2), an evolutionary conserved Ser/Thr protein kinase found in all eukaryotes, during abscisic acid (ABA) signaling and drought stress tolerance. For instance CK2 has a pivotal role in the regulation of ABA signaling through its action on *Zea mays* OST1/SnRK2.6 (Open Stomata 1/Sucrose Non Fermenting Kinase 2.6) a well-known Ser/Thr protein kinase involved in ABA signaling (Kulik et al., 2011). Furthermore, CK2-dependent phosphorylation enhances the stabilization and degradation of targeted proteins. CK2 is thought to be a housekeeping kinase which finely controls ABA signaling by mediating dynamic protein turnover. Another protein, this time involved in genome reprogramming during ABA signaling was identified by Li et al. They present new evidence showing interrelation between the transcription factor NAC072 and ABA responsive element binding factor ABF3, a positive regulator of ABA responsive gene expression. Interestingly, these two proteins could cooperate or act antagonistically, revealing a dual function of NAC072. ABA and other phytohormones could cooperate to orchestrate plant responses to stress. The research article of Jia and Li reports an example of this process known as hormone crosstalk in Arabidopsis, where ABA and ethylene accelerate senescence. The authors show that the suppression of phospholipase D (PLD) retards ethylene-promoted senescence (as previously reported for the ABA-promoted senescence) through an elusive mechanism resulting in the modification of plastidic lipid metabolism and maintenance of plasma membrane integrity. Lovieno et al. using a RNA sequencing approach, report on global transcriptomic changes associated with drought and rehydration in tomato. Transcriptomic analyses together with physiological measurements, quantification of metabolites and biometric parameters, yielded promising candidate genes and that could be used as specific physiological markers of plant drought response. Using a transgenic approach, two studies have functionally validated the role of two transcription factors during drought stress in rice and in soybean (*Glycine max*, Gm). Jiang et al. improved rice drought tolerance by overexpressing the *Arabidopsis thaliana* transcription factor WRKY57 (AtWRKY57) which was previously shown to enhanced drought resistance in Arabidopsis (Jiang et al., 2012). AtWRKY57 is a positive regulator of drought responses and appears to be a potential candidate gene for crop improvement but, as reported by the authors the effect on plant productivity should be analyzed to validate this strategy. However, Zhang et al. show that GmZFP3 (*Gl. max* Zinc Finger Protein 3) negatively regulates drought responses when overexpressed in Arabidopsis. GIGANTEA (GI), a plant specific nuclear protein, is a key component of flowering time regulation but is also known to be involved in a multitude of physiological responses. Finally, Li et al. report that mutation of *Oryza sativa* GI confers tolerance to osmotic stress and regulates transcript abundance of some gene encoding ABA-induced proteins. Tolerance to salinity and high temperature is also reviewed by HanumanthaRao et al. who presented an integrative view of mungbean responses from physiological, biochemical, and molecular aspects to agronomical perspectives and field management practices. Lastly, although Boron is considered as an essential micronutrients for plants, abnormal concentrations can be toxic and limit crop productivity. Fang et al. report that boron could alter calcium signaling and actin filament organization thus impacting on plant development (*Malus domestica* pollen tube growth). In addition to their essential function in plant primary metabolism, several molecules have now been considered as signal molecules. For instance, the signaling function of sugars has become the focus of numerous research efforts. Nguyen et al. using transgenic plants overexpressing sucrose synthase, reveal a novel sugar signaling pathway controlling pronounced phenotypic changes in tobacco. We can expect that future research will confirm the role of sugars throughout all stages of the plant's life cycle since sugar-signaling pathways could interact with other stimuli such as phytohormones or light (Smeekens, 2000). Lipids could also be considered at the interface of plant stress response and cellular primary metabolism. Levels of very long chain fatty acids (>18C, VLCFA) are known to be modified under stressful conditions. Based on preexisting data, De Bigault Du Grandrut and Cacas investigate through three scenarios, the concept that these VLCFA could also participate in stress signaling pathways. The authors proposed a model depicting the hypothetic role of VLCFA in a very informative and synthetic figure. Small molecule such as the diatomic gas carbon monoxide (CO), widely considered as detrimental, also emerges as signaling molecule in plants. The review of Wang and Liao provides brief update on its role in growth, development and abiotic stress tolerance. CO has a positive effects on salt or heavy metal stresses in relation with other signaling molecules, such as phytohormones or NO and ROS. Last but not least, research conducted by Kim et al. reveals links between N metabolism and epigenetics (gene methylation). They report that ammonium treatment inhibits chromomethylase 3-mediated methylation of the gene encoding one of the two nitrate reductase isoform (Nia2) in Arabidopsis. These results bring new insights concerning the regulation of nitrate assimilation and its signaling properties and open interesting perspectives for the role of epigenetics in plant responses to stress.

Biotic stresses also cause major losses in crop productivity. Deciphering mechanisms involved in plant defense to pathogens will help to develop breeding and biotechnological strategies for crop protection. Durian et al. provide an overview of the Protein Phosphatase 2A (PP2A) functions, a crucial component that controls pathogenic responses in various plant species. The authors describe, in an exhaustive manner, the connections of these multifunctional enzymes with the signaling pathway that controls plant immunity, cell death and more globally primary and secondary metabolism. Using an elegant biochemical and targeted approach, Sheikh et al. reveal a post-translational regulation of AtWKRY46 transcription factor by a MAPK3-dependent phosphorylation that mediates the stability of this transcription factor after PAMP elicitation in Arabidopsis. It is noteworthy that knowledge about plant molecular responses to biotic stress is obtained from model plants as well as from cultivated species. Thus, this topic reports the identification of regulators of plant immunity in different economically important crops. In soybean, Cheng et al. report the characterization of a novel member of the isoflavone reductase gene family, GmIFR, regulated by phytohormones (SA, ET, ABA) and involved in the resistance to the oomycete *Phytophthora sojae*. Using a quite similar functionally transgenic approach, Dai et al. have identified and characterized a new defense protein PR4-1 from a wild chinese grape *Vitis pseudoreticula*. When overexpressed in *Vitis vinifera*, it improved tolerance to powdery mildew. RNAseq methodology is now widely used to investigate gene expression in response to microbes in non-model species. Based on the analysis of the differentially expressed genes, Gao et al. propose a model illustrating the main molecular responses and gum polysaccharide formation in peach tree (*Prunus persica*) infected by *Lasiodiplodia theobromae*, the fungal agent of peach tree gummosis.

Yuan et al. compare by a RNAseq approach two symbiotic systems with notable different nodulation phenotypes in soybean roots. Many of the differentially expressed genes identified are related to plant immunity and could explain the different nodulation phenotypes. Their work highlights the delicate balance between beneficial and detrimental effects of microbes and, as written by the authors, it “sheds new light on the host legume control of nodulation specificity.”

Crosstalk between abiotic and biotic stress responses are the last aspect developed in this topic. Comparative approaches can be interesting to test the hypotheses of common signaling pathways and of physiological responses that are subject to pleiotropic gene action. In a mini-review article, Ranty et al. provide a broad perspective on the role of Ca ^2+^ in plant responses to abiotic and biotic stress. The specific effects of this ubiquitous second messenger and the role of calcium sensor proteins are discussed. The authors put forward hypotheses to explain one crucial question of cell signaling: how are signals perceived and how do cells respond spatially and temporally to these signals to program a specific response at the organism level? More specifically, Sinha et al. have examined the transcriptome dynamics in chickpea plants exposed to a combination of water deficit stress and *Ralstonia solanacearum* infection and have identified a set of genes uniquely expressed in response to combined stress. Also comparing pathogenic and drought stress but focusing on the analysis of redox homeostasis and the role phytohormones, Cui et al. show that co-stress by virus and drought had much severer effects than single stress in *V. vinifera*. Finally, Singh and Jha have studied crosstalk between salt stress and the bacteria *Bacillus licheniformis* HSW-16. They report biochemical and physiological characterization associated with bacteria-induced systemic tolerance to salt stress.

In conclusion, this Research Topic provides new results and reviews on the signaling pathways induced by abiotic and biotic environmental changes such as drought, high salinity, nutrient, pollutants, or microbial attacks. We think that the diversity of methodology, biological models, and physiological context used to decipher and understand these complex molecular mechanisms at the whole plant level will allow the development of sustainable practices and re-orientation of agricultural managements to improve stress tolerance in crops.

## Author contributions

All authors listed have made a substantial, direct and intellectual contribution to the work, and approved it for publication.

### Conflict of interest statement

The authors declare that the research was conducted in the absence of any commercial or financial relationships that could be construed as a potential conflict of interest.
